# Recent grip strength change and subsequent cognition after accounting for body mass index and waist circumference

**DOI:** 10.21203/rs.3.rs-11130121/v1

**Published:** 2026-10-16

**Authors:** Shenao Xi, Ting Gong, Huiyu Luo

## Abstract

Grip strength and cognition often decline together in later life, but it remains unclear whether recent change in strength provides information about subsequent cognition beyond current cognitive status and concurrent body-size change. We analysed 2,405 adults aged 60 years or older from the China Health and Retirement Longitudinal Study, contributing 3,594 person-period observations across two temporally ordered intervals. Generalized estimating equations related 2-year grip-strength change to cognition at the following wave while adjusting for cognition at the end of the change interval, starting grip strength, body mass index (BMI) level and change, and demographic, clinical and behavioural covariates. Each 5-kg greater grip-strength change was associated with a 0.149-point higher subsequent cognitive score (95% CI 0.046–0.252). Estimates were similar when waist circumference replaced BMI, under strict repeated-measurement quality criteria, after excluding absolute changes > 15 kg, and with inverse-probability weighting. There was no clear effect modification by sex or age. The association was modest and should be interpreted as a longitudinal marker rather than evidence of a causal effect or clinical screening threshold.

### Introduction

Grip strength is a simple measure of neuromuscular function and an established marker of health in later life. Prospective studies and meta-analyses have associated weaker grip strength with cognitive decline and dementia, while coordinated analyses suggest that within-person changes in strength and cognition often track together [4–6]. These observations support grip strength as a marker of biological and functional aging, but they do not by themselves establish the temporal sequence or clinical magnitude of the association.

Evidence from CHARLS has expanded rapidly. Baseline grip strength has was associated with later cognition [7], recent cross-lagged analyses have reported bidirectional associations between strength and cognitive domains [8, 9], trajectory analyses have related absolute and relative strength patterns to cognitive performance [10], and year-to-year strength variability has been associated with cognitive trajectories [11]. Related work has also examined dynapenia together with abdominal obesity [12]. The remaining question is therefore narrower than whether grip strength and cognition are associated.

A recent directional change can be aligned explicitly before a later cognitive assessment. Conditioning on cognition measured at the end of the change interval separates the analysis from a concurrent change-change comparison and asks whether preceding strength change provides information about the next cognitive assessment among individuals with the same current cognitive score. Comparing strength change with time-aligned changes in BMI and waist circumference further distinguishes neuromuscular change from changes in measured body size. BMI and waist circumference are body-size measures rather than direct measures of adipose tissue, particularly in older adults, and should not be treated as interchangeable with adiposity [13, 14].

We therefore evaluated two repeated, temporally ordered intervals in CHARLS. We hypothesized that a more favorable recent grip-strength change would be associated with better subsequent cognitive performance after adjustment for cognition at the end of the change interval, starting grip strength, and concurrent changes in BMI or waist circumference. We also examined the magnitude of the association, potential sex and age heterogeneity, the fixed 5-kg scale, unusually large changes, within-session repeatability, nonlinear form, unequal cognitive follow-up intervals, and selection into the longitudinal risk sets.

### Results

## Participants and analytic selection

The source cohort included 7,290 adults aged 60 years or older. The fully adjusted analysis included 2,405 participants contributing 3,594 person-period observations: 2,157 from the 2011–2013 to 2015 sequence and 1,437 from the 2013–2015 to 2018 sequence. Included participants were younger than those not included (mean 65.99 vs 69.43 years), had higher baseline grip strength (32.07 vs 27.30 kg) and cognition (15.01 vs 13.00 points), and had fewer depressive symptoms (8.21 vs 9.53 CES-D points) (Table 1). The inclusion rate declined from 41.7% at ages 60–64 years to 4.9% at age 80 years or older.

Table 1
Baseline characteristics according to inclusion in the longitudinal analytic sample

| Characteristic | Included (n = 2,405) | Not included (n = 4,885) | SMD |
|---|---|---|---|
| Age, years | 65.99 (5.01) | 69.43 (7.38) | -0.546 |
| Grip strength, kg | 32.07 (9.33) | 27.30 (9.45) | 0.508 |
| Body mass index, kg/m2 | 23.12 (3.73) | 22.72 (4.06) | 0.103 |
| Waist circumference, cm | 85.38 (10.38) | 85.23 (10.66) | 0.014 |
| Global cognitive score | 15.01 (4.10) | 13.00 (5.04) | 0.436 |
| CES-D score | 8.21 (6.12) | 9.53 (6.59) | -0.208 |
| Female sex | 984 (40.9%) | 2643 (54.1%) | -0.266 |
| Education low | 982 (40.8%) | 3201 (65.6%) | -0.512 |
| Education middle | 1233 (51.3%) | 1352 (27.7%) | 0.497 |
| Education high | 190 (7.9%) | 329 (6.7%) | 0.045 |
| Married or partnered | 2040 (84.8%) | 3619 (74.2%) | 0.266 |
| Rural residence | 1503 (62.5%) | 2873 (58.8%) | 0.075 |
| Hypertension | 763 (31.7%) | 1713 (35.7%) | -0.084 |
| Diabetes | 194 (8.1%) | 354 (7.4%) | 0.024 |
| Heart disease | 371 (15.4%) | 809 (16.9%) | -0.040 |
| Stroke | 66 (2.7%) | 245 (5.1%) | -0.121 |
| Cancer | 24 (1.0%) | 46 (1.0%) | 0.004 |
| Former smoker | 299 (12.4%) | 515 (11.1%) | 0.041 |
| Current smoker | 835 (34.7%) | 1203 (26.0%) | 0.191 |
| Alcohol use | 855 (35.6%) | 1300 (26.9%) | 0.186 |

Values are mean (SD) or n (%). Percentages use nonmissing denominators. SMD indicates standardized mean difference. Missing data in the source cohort were present for grip strength (n = 1,540), BMI (n = 1,523), waist circumference (n = 1,525), cognition (n = 2,459), CES-D (n = 598), education (n = 3), marital status (n = 6), chronic diseases (n = 72–112), smoking (n = 256), and alcohol use (n = 61).

### Primary longitudinal association

In the fully adjusted BMI model, each 5-kg greater grip-strength change over the preceding 2-year interval was associated with a 0.149-point higher subsequent cognitive score (beta = 0.149, 95% CI 0.046–0.252;

P = 0.005). Starting grip strength was also positively associated with subsequent cognition (beta = 0.211 per 5 kg, 95% CI 0.102–0.321; P < 0.001). Neither concurrent BMI change (beta = 0.033 per kg/m2, 95% CI -0.035 to 0.101; P = 0.343) nor starting BMI (beta = 0.019, 95% CI -0.020 to 0.057; P = 0.341) was independently associated with the outcome (Table 2). The primary effect was statistically detectable but small: a 5-kg difference corresponded to approximately 0.15 points on the 30-point cognitive score.

Table 2
Selected coefficients from the main longitudinal models for subsequent cognitive function

| Body-size model | Predictor | Beta | 95% CI | P value |
|---|---|---|---|---|
| BMI | Grip-strength change, per 5 kg | 0.149 | 0.046 to 0.252 | 0.005 |
| BMI | Starting grip strength, per 5 kg | 0.211 | 0.102 to 0.321 | < 0.001 |
| BMI | BMI change, per kg/m2 | 0.033 | -0.035 to 0.101 | 0.343 |
| BMI | Starting BMI, per kg/m2 | 0.019 | -0.020 to 0.057 | 0.341 |
| Waist | Grip-strength change, per 5 kg | 0.173 | 0.070 to 0.277 | 0.001 |
| Waist | Waist change, per 5 cm | 0.060 | -0.044 to 0.164 | 0.261 |

Gaussian GEE with exchangeable working correlation and robust standard errors. The BMI model included 3,594 person-periods from 2,405 participants; the waist model included 3,569 person-periods from 2,396 participants. Models adjust for current cognition, age, sex, education, marital status, rural residence, hypertension, diabetes, heart disease, stroke, cancer, baseline CES-D score, smoking, alcohol use, and observation period. BMI, body mass index.

### Waist circumference and direct exposure contrasts

Replacing BMI with waist circumference yielded a similar grip-strength estimate (beta = 0.173 per 5 kg, 95% CI 0.070–0.277; P = 0.001). Waist-circumference change (beta = 0.060 per 5 cm, P = 0.261) and starting waist circumference (P = 0.957) were not independently associated with subsequent cognition. Standardized point estimates were larger for grip-strength change than for BMI change (0.216 vs 0.087) and waist-circumference change (0.242 vs 0.111), but direct coefficient contrasts were not statistically significant (P = 0.204 and P = 0.220, respectively). The data therefore did not establish that grip-strength change was more important than either body-size change measure. Selected coefficients from both body-size models are shown in Table 2.

### Measurement sensitivity analyses

The fully adjusted estimate was nearly unchanged after stringent repeat-measurement quality restrictions (3,235 observations from 2,198 participants; beta = 0.150, 95% CI 0.037–0.264; P = 0.009). Excluding 172 person-periods with absolute grip-strength change greater than 15 kg left 3,422

observations from 2,316 participants and produced a larger estimate (beta = 0.243, 95% CI 0.118–0.367; P < 0.001). The primary association was therefore not driven by the most extreme observed changes (Table 3 and Fig. 2).

Table 3
Sensitivity analyses of grip-strength change

| Analysis | Outcome | Scale | Beta | 95% CI | P value |
|---|---|---|---|---|---|
| Primary fully adjusted BMI model | Subsequent cognition | Per 5 kg | 0.149 | 0.046 to 0.252 | 0.005 |
| Waist circumference instead of BMI | Subsequent cognition | Per 5 kg | 0.173 | 0.070 to 0.277 | 0.001 |
| Winsorized at 1st-99th percentiles | Subsequent cognition | Per 5 kg | 0.163 | 0.055 to 0.271 | 0.003 |
| Independence working correlation | Subsequent cognition | Per 5 kg | 0.162 | 0.058 to 0.265 | 0.002 |
| Interval-specific IPCW | Subsequent cognition | Per 5 kg | 0.156 | 0.046 to 0.265 | 0.006 |
| IPCW truncated 1st-99th percentiles | Subsequent cognition | Per 5 kg | 0.158 | 0.048 to 0.268 | 0.005 |
| Strict repeated-trial QC | Subsequent cognition | Per 5 kg | 0.150 | 0.037 to 0.264 | 0.009 |
| Exclude absolute change > 15 kg | Subsequent cognition | Per 5 kg | 0.243 | 0.118 to 0.367 | < 0.001 |
| Standardized within sex and period | Subsequent cognition | Per SD | 0.224 | 0.072 to 0.377 | 0.004 |
| Annualized cognitive-change model | Cognitive change/year | Per 5 kg | 0.071 | 0.025 to 0.117 | 0.002 |

Estimates use the fully adjusted covariate set unless indicated. The standardized and annualized models use different coefficient scales and should not be compared numerically with the per-5-kg subsequent-cognition estimates. IPCW, inverse-probability-of-censoring weighting; QC, quality control.

### Sex, age, scale, and period

The point estimate was larger in men (beta = 0.189 per 5 kg, 95% CI 0.068–0.310) than in women (beta = 0.079, 95% CI -0.077 to 0.235), but the grip-change-by-sex interaction was not statistically significant (P = 0.231). Estimates increased numerically between ages 60 and 80 years, but the continuous age interaction was also inconclusive (P = 0.177). When grip-strength change was standardized within sex and observation period, the association remained evident (beta = 0.224 cognitive points per SD, 95% CI 0.072–0.377; P = 0.004). Sex- and age-specific estimates are shown in Table 4.

Table 4
Sex- and age-specific estimates

| Characteristic | Level | Beta per 5 kg | 95% CI | P value |
|---|---|---|---|---|
| Sex | Male | 0.189 | 0.068 to 0.310 | 0.002 |
| Sex | Female | 0.079 | -0.077 to 0.235 | 0.321 |
| Age | 60 years | 0.084 | -0.052 to 0.221 | 0.226 |
| Age | 65 years | 0.143 | 0.040 to 0.246 | 0.007 |
| Age | 70 years | 0.201 | 0.071 to 0.331 | 0.002 |
| Age | 75 years | 0.260 | 0.066 to 0.454 | 0.009 |
| Age | 80 years | 0.318 | 0.049 to 0.588 | 0.021 |
| The formal grip-change-by-sex interaction P value was 0.231; the grip-change-by-age interaction P value was 0.177. Stratum-specific statistical significance should not be interpreted as evidence of heterogeneity. | | | | |

The period-specific estimate was 0.099 (95% CI -0.012 to 0.210; P = 0.079) for 2011–2013 change predicting 2015 cognition and 0.266 (95% CI 0.093–0.439; P = 0.003) for 2013–2015 change predicting 2018 cognition; evidence for heterogeneity was inconclusive (Pinteraction = 0.074). After annualizing subsequent cognitive change, the pooled association remained positive (beta = 0.071 points/year per 5 kg, 95% CI 0.025–0.117; P = 0.002), and the period interaction weakened (Pinteraction = 0.133). Period-specific and annualized estimates are summarized in Table 5.

Table 5
Period-specific and annualized estimates

| Period | Outcome | Beta per 5 kg | 95% CI | P value |
|---|---|---|---|---|
| 2011–2013 change to 2015 cognition | Subsequent cognition | 0.099 | -0.012 to 0.210 | 0.079 |
| 2013–2015 change to 2018 cognition | Subsequent cognition | 0.266 | 0.093 to 0.439 | 0.003 |
| 2011–2013 change to 2015 cognition | Annualized change/year | 0.053 | -0.002 to 0.108 | 0.059 |
| 2013–2015 change to 2018 cognition | Annualized change/year | 0.110 | 0.048 to 0.172 | < 0.001 |
| Interaction P values were 0.074 for subsequent cognition and 0.133 for annualized cognitive change. | | | | |

## Other sensitivity analyses

Results were similar after 1st-99th percentile winsorization (beta = 0.163, 95% CI 0.055–0.271; P = 0.003) and under an independence working correlation (beta = 0.162, 95% CI 0.058–0.265; P = 0.002). Interval-specific inverse-probability weighting yielded beta = 0.156 (95% CI 0.046–0.265; P = 0.006), and truncating weights at the 1st and 99th percentiles yielded beta = 0.158 (95% CI 0.048–0.268; P = 0.005). A natural-spline model had lower QIC than the linear specification, but the joint test for nonlinearity was inconclusive (Wald chi-square = 4.81, 2 df; P = 0.090); the linear effect therefore remained the primary summary. In the broader baseline-cohort weighting analysis, the estimate attenuated to 0.099 (95% CI -0.023 to 0.221; P = 0.110), consistent with limited overlap and uncertainty about generalizability to older, less healthy participants who could not form longitudinal intervals.

### Measurement-quality audit

Within-session repeatability was generally high. Median absolute differences between repeated trials were approximately 1.5-2.0 kg, and 0.6%-1.3% of hand-specific trial pairs differed by more than 10 kg. In the broader repeated-grip dataset, 93.4% of person-periods with absolute change greater than 15 kg showed concordant directional changes in both hands, and 89.1% showed changes greater than 10 kg in both hands. Thus, unusually large changes were not predominantly attributable to unilateral missingness or a single discrepant trial. The measurement-quality audit is summarized in Table 6.

Table 6
Measurement-quality audit

| Measure | Wave or subset | Result |
|---|---|---|
| Valid primary grip measure | 2011 | 5,750 participants |
| Valid primary grip measure | 2013 | 4,615 participants |
| Valid primary grip measure | 2015 | 4,743 participants |
| Strict QC grip measure | 2011 | 5,381 participants |
| Strict QC grip measure | 2013 | 4,297 participants |
| Strict QC grip measure | 2015 | 4,369 participants |
| Within-session absolute trial difference | All waves | Median 1.5-2.0 kg; >10 kg in 0.6%-1.3% |
| Absolute 2-year change > 15 kg | Broader repeated-grip data | 396 person-periods |
| Concordant change direction in both hands | Among > 15-kg changes | 93.4% |
| Both hands changed > 10 kg | Among > 15-kg changes | 89.1% |
| Strict QC required two valid trials per hand, within-hand trial differences no greater than 10 kg, averaging within each hand, and selecting the higher hand mean. | | |

## Discussion

In this longitudinal analysis, a more favorable recent grip-strength change was associated with better subsequent cognitive performance after cognition at the end of the change interval, starting strength, concurrent BMI change, and a broad set of baseline covariates were taken into account. The estimate remained positive when BMI was replaced by waist circumference and across analyses addressing within-session repeatability, unusually large changes, relative scaling, unequal cognitive follow-up intervals, working correlation, and interval-specific censoring. The association was modest: a 5-kg difference corresponded to about 0.15 points on a 30-point cognitive score.

The contribution of this study is the temporal alignment, not a new claim that grip strength and cognition are associated. Earlier CHARLS studies have evaluated baseline strength, bidirectional cross-lagged relationships, long-term strength trajectories, and year-to-year variability [7–11]. We instead defined a preceding 2-year directional change, conditioned on cognition measured at the end of that interval, and related the change to a later cognitive assessment while simultaneously modeling time-aligned change in BMI or waist circumference. This design reduces, but does not eliminate, concerns that the observed association reflects only baseline differences or concurrent cognitive status.

The measurement-quality analyses address an important limitation of difference scores. Large 2-year changes were common enough to raise concern, but they were usually bilateral and directionally concordant. Requiring consistent repeated trials produced an estimate nearly identical to the primary result, and removing absolute changes greater than 15 kg increased rather than attenuated the estimate. These findings suggest that a few extreme measurements did not create the association. They do not prove that all observed change was physiological; effort, health events, testing conditions, and regression to the mean may still contribute.

Sex-specific and age-specific estimates should be interpreted from the interaction tests rather than from whether a stratum-specific P value crossed 0.05. Although the point estimate was larger in men and increased with age, neither interaction was conclusive. The standardized analysis also showed that the result was not dependent on assigning the same relative meaning to a 5-kg change across sex and period. The pooled estimate therefore remains the most defensible summary.

BMI and waist circumference are imperfect measures of body composition. Their null coefficients in these models do not show that adipose tissue is unrelated to cognitive aging, and the nonsignificant direct Wald contrasts do not establish that grip-strength change is the more important exposure. The current data support only the narrower conclusion that the grip-strength association persisted after concurrent changes in measured body size were taken into account.

Grip strength may integrate central motor drive, peripheral neuromuscular function, nutrition, physical activity, vascular health, inflammation, and multimorbidity. These pathways may influence cognition, but declining cognition can also reduce activity and test performance. Conditioning on current cognition clarifies temporal ordering but cannot remove time-varying confounding or reverse causation that began before the observed interval. Grip-strength change is therefore best viewed here as a longitudinal marker of multisystem aging rather than a demonstrated causal target.

The attenuation in the broad baseline-cohort weighting analysis is also important. Participants who could form complete longitudinal intervals were younger, stronger, and cognitively healthier, and those aged 80 years or older were poorly represented. Interval-specific weighting, which addressed subsequent cognitive missingness within eligible change intervals, preserved the primary estimate. The broader analysis addresses a more demanding generalizability question and had poor overlap. Together, these findings support the internal longitudinal association while limiting extrapolation to the oldest and least healthy members of the baseline cohort.

Strengths include two repeated temporal sequences, objective repeated grip and anthropometric measurements, conditioning on cognition at the end of the exposure interval, adjustment for starting levels and concurrent body-size change, formal direct coefficient contrasts, and targeted measurement-quality and selection analyses. The principal limitations are the observational design, residual and time-varying confounding, measurement error in difference scores, non-equivalence of some cognitive items across waves, unequal follow-up intervals, complete-case selection, limited representation of adults aged 80 years or older, and lack of direct body-composition measures. The 0–30 cognitive score is not a clinical diagnostic instrument, and the observed 0.149-point difference should not be treated as a clinically meaningful threshold. Multiple complementary analyses were performed without multiplicity adjustment and should be considered supportive.

Recent grip-strength change was modestly associated with subsequent cognitive performance after prior cognition and concurrent changes in BMI or waist circumference were taken into account. The result was not driven by the largest observed changes or poor repeat-measurement quality and showed no clear heterogeneity by sex or age. Because the effect was small and the study remains vulnerable to selection, measurement, and reverse-causation biases, grip-strength change should be interpreted as a population-level marker of longitudinal functional aging rather than a stand-alone cognitive screening threshold or a proven intervention target.

### Methods

## Study design and population

CHARLS is a nationally representative longitudinal study of Chinese adults aged 45 years or older and their spouses [1, 2]. We used the 2011, 2013, 2015, and 2018 waves of the Harmonized CHARLS data [3]. The source cohort comprised 7,290 respondents who participated in the 2011 baseline survey and were aged 60 years or older. The present analysis used two person-period records when the required exposure, outcome, and covariate data were available. The final fully adjusted dataset included 2,405 participants and 3,594 person-period observations. Reporting follows the STROBE recommendations [15].

The design separated exposure and outcome periods (Fig. 1). Interval 1 used grip-strength and body-size change from 2011 to 2013, cognition in 2013 as the current cognitive score, and cognition in 2015 as the subsequent outcome. Interval 2 used change from 2013 to 2015, cognition in 2015 as the current score, and cognition in 2018 as the subsequent outcome.

## Grip strength and change variables

Trained interviewers measured grip strength twice in each hand with a dynamometer. Harmonized hand-specific values represented the higher valid trial for that hand. Values greater than 0 and no more than 80 kg were considered valid. The primary wave-specific measure was the higher value from the two hands. Grip-strength change was calculated as end minus start of each 2-year interval; positive values therefore indicated gain or a smaller decline, and negative values indicated decline. The principal coefficient was scaled per 5-kg greater change. Starting grip strength, also scaled per 5 kg, was included to distinguish recent change from the participant's initial level.

## Body mass index and waist circumference

BMI was calculated from measured weight and height and restricted to 12–60 kg/m2. Waist circumference was restricted to 50–150 cm. For each interval, change was calculated as end minus start. The primary model included BMI change and starting BMI. A parallel model replaced both BMI terms with waist-circumference change and starting waist circumference. Throughout the manuscript, these variables are described as body-size measures rather than direct measures of adiposity.

## Cognitive assessment

Global cognition combined episodic memory, serial subtraction, temporal orientation, and figure copying. Component scores were summed to a 0–30 score, with higher scores indicating better performance. The primary outcome was the score at the wave after each exposure interval. Cognition measured at the end of the exposure interval was included as a covariate, so the coefficient for grip-strength change represented its association with later cognition among participants with the same current cognitive score.

## Covariates

The fully adjusted model included baseline age, sex, education, marital status, rural residence, hypertension, diabetes, heart disease, stroke, cancer, baseline 10-item Center for Epidemiologic Studies Depression score, smoking status, alcohol use, and observation period. Education was categorized as low, middle, or high; smoking as never, former, or current; and marital status as married or partnered versus not married.

## Statistical analysis

We summarized baseline characteristics according to inclusion in the fully adjusted longitudinal analysis and used standardized mean differences to describe selection. The primary analysis used a Gaussian generalized estimating equation with an exchangeable working correlation structure and robust standard errors, clustering repeated person-period observations by participant. The primary parameter was the adjusted difference in subsequent cognitive score per 5-kg greater grip-strength change.

We performed complementary analyses selected to address interpretation and measurement concerns. Standardized models placed grip, BMI, and waist changes on SD scales and used covariance-based Wald contrasts to compare coefficients. Effect modification was tested with grip-change-by-sex and grip-change-by-age interactions; age was centered at 65 years and scaled per 5 years. We also standardized grip-strength change within sex and period to evaluate a relative rather than fixed absolute scale.

Measurement sensitivity analyses (1) winsorized grip-strength change at the 1st and 99th percentiles in the fully adjusted sample; (2) required two valid trials in each hand, excluded hand-specific trial pairs differing by more than 10 kg, averaged the two trials within each hand, and then selected the higher hand mean; and (3) excluded person-periods with absolute change greater than 15 kg. The 10-kg repeatability rule was a data-informed quality restriction, approximately corresponding to the extreme 1% of within-session trial differences, not a clinical cutoff.

Additional analyses used an independence working correlation, period-specific models, annualized subsequent cognitive change to account for the 2- and 3-year intervals from current to subsequent cognition, and a 3-degree-of-freedom natural spline with a robust joint Wald test for departure from linearity. Interval-specific stabilized inverse-probability-of-censoring weights addressed missing subsequent cognition among records eligible to define a change interval; weights were also truncated at the 1st and 99th percentiles. A broader baseline-cohort selection-weighting analysis was retained as a generalizability sensitivity analysis because overlap was poor among the oldest participants. Tests were two-sided, and complementary analyses were interpreted by effect size, direction, and confidence intervals rather than statistical significance alone. Analyses were conducted in R with geepack and related packages.

## Declarations

#### Ethics approval and consent to participate

CHARLS was approved by the Institutional Review Board of Peking University (IRB00001052-11015 for the main household survey and IRB00001052-11014 for biomarker collection). All methods were carried out in accordance with relevant guidelines and regulations, and written informed consent was obtained from all participants. The present study used de-identified secondary data and involved no direct participant contact.

#### AI-assisted writing disclosure

ChatGPT (OpenAI) was used to assist language editing, manuscript organization, and discussion of statistical analysis options. All statistical analyses were executed by the authors in R, and all analytical choices, outputs, references, interpretations, and final text were reviewed and verified by the authors.

#### Data availability

CHARLS data are available to registered users through the CHARLS data portal, subject to the applicable data-use agreement.

#### Acknowledgements

This analysis uses the Harmonized CHARLS dataset and Codebook, Version D, developed by the Gateway to Global Aging Data. The Harmonized CHARLS was supported by the National Institute on Aging (R01 AG030153, RC2 AG036619, and R03 AG043052).

#### Author contributions

Shenao Xi: conceptualization, methodology, data curation, formal analysis, visualization, and writing-original draft. Ting Gong: conceptualization, methodology, validation, formal analysis, interpretation, writing-original draft, and writing-review and editing. Huiyu Luo: conceptualization, supervision, project administration, validation, interpretation, and writing-review and editing. All authors read and approved the manuscript.

#### Funding

This research received no external funding.

#### Competing interests

The authors declare no competing interests.

## Figures

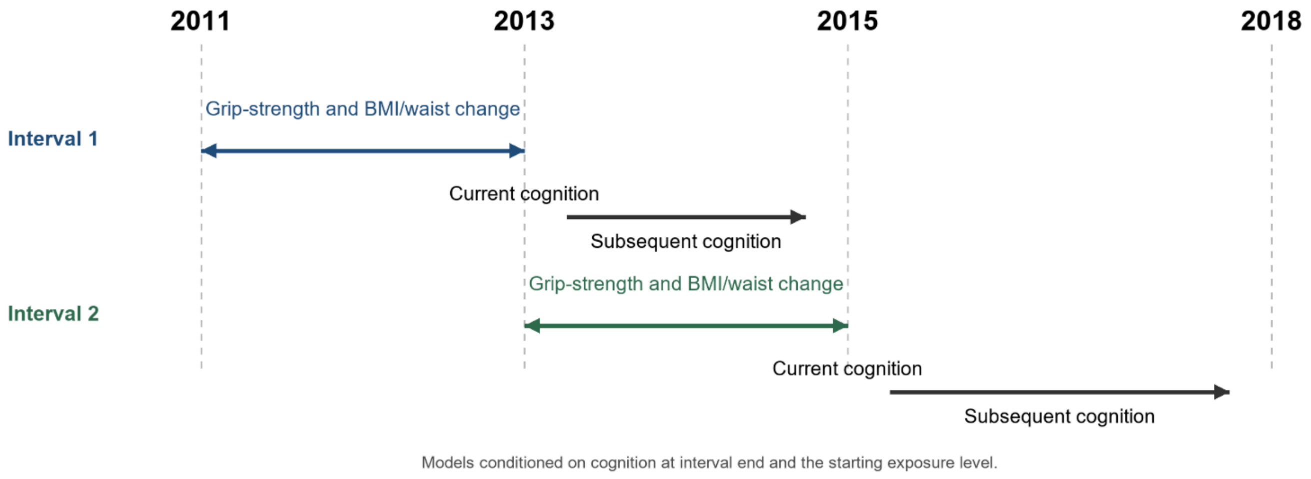

### Figure 1

Temporally ordered study design. Grip strength and BMI or waist circumference were measured over each 2-year change interval. Cognition measured at the end of the change interval was included as a covariate, and cognition at the next wave was the primary outcome.

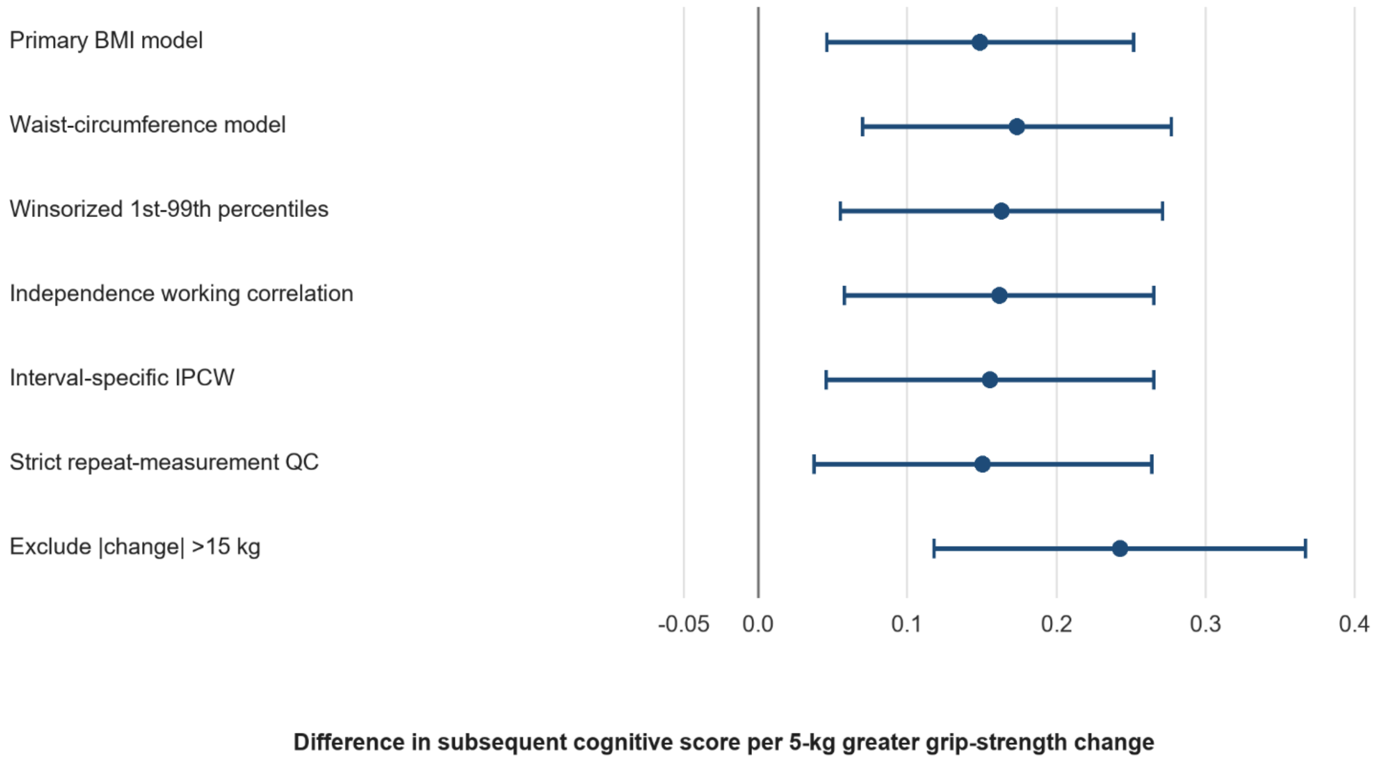

### Figure 2

Grip-strength change estimates in the primary and measurement sensitivity analyses. Points show adjusted differences in subsequent cognitive score per 5-kg greater grip-strength change; bars show

95% confidence intervals. The figure excludes analyses with a different coefficient scale. IPCW, inverse-probability-of-censoring weighting; QC, quality control.
